# Supplementary material for: Recovery of Scots Pine Seedlings from Long-Term Zinc Toxicity
Source: Plants (Basel). 2024 Aug 11;13(16):2227. doi: 10.3390/plants13162227 (PMC11359686; doi:10.3390/plants13162227)
Supplement: Supplementary file 1 [file plants-13-02227-s001.zip › Table S2.pdf]

Table S2. The dynamics of nutrient content in the organs of Scots pine seedlings throughout the experiment.

Variant	Initial point	Day of the experiment								
		1st	3rd	5th	7th	10th	14th	17th	21st	28th
Zinc, µmol/g DW										
Roots										
Control	2.85 ± 0.34	3.27 ± 0.33	2.55 ± 0.18	2.79 ± 0.19	3.32 ± 0.48	3.30 ± 0.38	2.77 ± 0.32	2.49 ± 0.25	2.49 ± 0.26	3.54 ± 0.41
150 µM Zn	61.9 ± 4.05×	54.7 ± 3.34×	55.8 ± 4.01×	52.2 ± 4.85×	45.4 ± 2.67×	44.7 ± 4.06×	46.0 ± 2.76×	40.6 ± 2.82×	36.6 ± 2.98×	50.7 ± 3.89×
Recovery	61.9 ± 4.05×	57.6 ± 2.15×	39.0 ± 1.90×	34.6 ± 2.19×	31.9 ± 2.45×	22.2 ± 2.46×	16.7 ± 1.09×	13.0 ± 1.26×	14.7 ± 0.82×	10.8 ± 0.88*
Hypocotyls										
Control	1.46 ± 0.14	1.83 ± 0.33	1.67 ± 0.60	1.15 ± 0.24	1.40 ± 0.13	0.87 ± 0.15	0.94 ± 0.03	1.20 ± 0.13	0.92 ± 0.11	0.98 ± 0.05
150 µM Zn	12.3 ± 1.13*	11.1 ± 0.84*	10.9 ± 0.52×	10.1 ± 1.29×	14.6 ± 1.87×	8.21 ± 0.51*	11.4 ± 1.66×	11.4 ± 1.47*	9.48 ± 0.96×	11.1 ± 1.82×
Recovery	12.3 ± 1.13*	14.8 ± 1.31×	11.0 ± 2.80×	11.3 ± 2.20×	9.60 ± 0.74×	6.04 ± 0.97×	5.20 ± 0.40×	7.67 ± 1.44×	4.85 ± 0.50×	4.18 ± 0.89×
Cotyledons										
Control	1.47 ± 0.06	1.77 ± 0.04	2.10 ± 0.41	2.42 ± 0.46	1.50 ± 0.14	2.07 ± 0.19	1.65 ± 0.16	1.22 ± 0.11	1.64 ± 0.10	1.73 ± 0.41
150 µM Zn	9.64 ± 0.54×	11.2 ± 1.35×	10.7 ± 1.20×	12.4 ± 1.88*	11.0 ± 0.96×	11.7 ± 0.58×	12.0 ± 1.41*	9.31 ± 0.97×	11.8 ± 1.35×	12.1 ± 1.25*
Recovery	9.64 ± 0.54×	11.6 ± 0.74×	10.2 ± 0.44*	13.7 ± 1.12*	9.54 ± 1.05×	11.3 ± 0.64×	10.8 ± 0.45×	9.64 ± 1.20×	11.9 ± 0.77×	9.46 ± 0.46*
Needles										
Control	1.04 ± 0.05	1.10 ± 0.07	1.04 ± 0.06	1.04 ± 0.05	1.04 ± 0.06	1.01 ± 0.04	1.06 ± 0.08	0.93 ± 0.02	1.04 ± 0.06	1.15 ± 0.05
150 µM Zn	9.12 ± 0.42×	8.95 ± 0.67×	8.43 ± 0.74×	7.87 ± 0.49×	8.34 ± 0.61×	8.20 ± 0.41×	7.76 ± 0.36×	9.82 ± 0.91×	8.64 ± 0.48×	8.71 ± 0.24×
Recovery	9.12 ± 0.42×	8.88 ± 0.57×	8.24 ± 0.53×	8.42 ± 0.54×	8.11 ± 0.60×	7.58 ± 0.65×	5.81 ± 0.33×	6.11 ± 0.55×	5.65 ± 0.47×	4.14 ± 0.26*
Magnesium, µmol/g DW										
Roots										
Control	52.9 ± 3.97	55.6 ± 3.67	44.7 ± 2.46	45.7 ± 2.54	53.7 ± 3.90	51.0 ± 4.18	46.1 ± 3.56	48.9 ± 3.62	44.1 ± 2.96	52.0 ± 3.20
150 µM Zn	33.1 ± 2.40*	26.9 ± 1.86*	21.3 ± 1.37*	24.8 ± 1.94*	29.2 ± 3.30*	24.4 ± 2.40*	27.1 ± 1.62*	31.1 ± 2.62*	22.8 ± 1.17*	30.4 ± 1.62*
Recovery	33.1 ± 2.40*	29.0 ± 1.49*	29.0 ± 1.57*	29.8 ± 1.95*	44.0 ± 3.07×	36.5 ± 2.73*	36.6 ± 2.55*	49.5 ± 3.41	43.5 ± 1.38	51.9 ± 2.80
Hypocotyls										
Control	59.4 ± 3.53	63.0 ± 2.17	62.5 ± 4.03	64.4 ± 5.03	73.1 ± 3.76	55.4 ± 5.66	51.1 ± 1.59	59.2 ± 9.18	50.1 ± 3.09	46.3 ± 2.30
150 µM Zn	53.3 ± 3.33	49.7 ± 1.31*	51.4 ± 2.76	50.6 ± 4.06	62.8 ± 8.38	53.5 ± 6.51	51.6 ± 5.41	31.2 ± 3.05×	32.5 ± 3.96*	36.7 ± 4.64
Recovery	53.3 ± 3.33	60.6 ± 5.43	54.1 ± 4.53	60.3 ± 7.85	62.0 ± 4.25	52.8 ± 2.79	54.8 ± 2.00	57.3 ± 5.00	53.3 ± 4.67	44.2 ± 3.49
Cotyledons										
Control	171.9 ± 5.78	178.1 ± 8.21	180.9 ± 11.2	175.2 ± 10.2	185.7 ± 6.10	170.9 ± 10.7	192.8 ± 9.74	158.5 ± 3.92	187.3 ± 8.11	164.8 ± 8.28
150 µM Zn	166.1 ± 2.34	188.9 ± 9.89	184.1 ± 8.61	186.0 ± 18.2	181.2 ± 9.63	160.4 ± 7.06	201.8 ± 10.9	165.8 ± 9.57	168.9 ± 13.9	160.9 ± 11.5
Recovery	166.1 ± 2.34	182.3 ± 14.6	152.6 ± 6.00	202.6 ± 11.9	165.3 ± 11.1	184.8 ± 11.3	157.0 ± 4.79*	176.4 ± 7.5	179.3 ± 5.86	170.4 ± 7.90
Needles										
Control	78.1 ± 4.61	81.1 ± 4.00	73.8 ± 1.81	76.2 ± 2.41	82.9 ± 3.80	76.3 ± 4.80	81.4 ± 3.68	80.9 ± 3.23	76.0 ± 2.91	84.3 ± 4.81
150 µM Zn	57.6 ± 1.02*	55.5 ± 3.11*	53.0 ± 1.19*	55.5 ± 1.55*	57.4 ± 3.42*	51.0 ± 3.01*	56.5 ± 1.77*	57.7 ± 3.18*	49.0 ± 1.68*	53.2 ± 1.81×
Recovery	57.6 ± 1.02*	53.2 ± 2.09*	53.9 ± 2.13*	53.9 ± 3.50*	65.1 ± 3.85	63.2 ± 2.39*	63.3 ± 2.85*	73.1 ± 4.13	74.1 ± 2.58	77.4 ± 2.29
Iron, µmol/g DW										
Roots										
Control	53.6 ± 8.81	65.9 ± 5.50	57.0 ± 8.15	54.9 ± 4.82	47.4 ± 3.51	73.7 ± 9.22	99.1 ± 6.86	109.8 ± 19.9	103.0 ± 13.2	219.5 ± 25.9
150 µM Zn	37.5 ± 3.74	52.8 ± 4.50	47.5 ± 6.14	57.0 ± 1.49	58.7 ± 7.29	59.8 ± 3.51	76.2 ± 10.9	75.7 ± 7.93	92.9 ± 8.22	167.9 ± 35.7
Recovery	37.5 ± 3.74	47.8 ± 9.94	34.7 ± 3.84*	52.4 ± 4.95	43.2 ± 7.19	73.6 ± 19.8	74.4 ± 10.3	124.5 ± 19.2	130.3 ± 12.2	200.8 ± 17.7
Hypocotyls										
Control	1.63 ± 0.36	1.23 ± 0.30	0.98 ± 0.19	1.65 ± 0.30	2.19 ± 0.50	2.29 ± 0.57	0.36 ± 0.03	1.14 ± 0.19	1.06 ± 0.23	1.54 ± 0.31
150 µM Zn	1.27 ± 0.15	1.85 ± 0.44	2.40 ± 0.14*	1.93 ± 0.63	2.01 ± 0.26	1.72 ± 0.30	1.04 ± 0.33	2.04 ± 0.41	0.54 ± 0.25	2.02 ± 0.41
Recovery	1.27 ± 0.15	2.25 ± 0.40	1.44 ± 0.20	2.29 ± 0.87	2.14 ± 1.18	0.95 ± 0.34×	0.73 ± 0.19	2.49 ± 0.63	0.76 ± 0.21	1.24 ± 0.18
Cotyledons										
Control	1.65 ± 0.13	2.54 ± 0.47	1.91 ± 0.58	2.06 ± 0.29	3.41 ± 1.08	2.38 ± 0.71	4.21 ± 1.32	2.15 ± 0.76	2.28 ± 0.22	3.04 ± 1.39
150 µM Zn	1.74 ± 0.39	1.92 ± 0.25	0.97 ± 0.31	2.32 ± 0.60	1.60 ± 0.51	0.98 ± 0.27	2.49 ± 0.60	1.34 ± 0.14	5.29 ± 1.64	1.67 ± 0.45
Recovery	1.74 ± 0.39	2.26 ± 0.20	1.57 ± 0.31	1.89 ± 0.48	2.51 ± 0.72	1.42 ± 0.34	1.89 ± 0.22	3.37 ± 0.57	3.63 ± 0.82	2.42 ± 0.53
Needles										
Control	2.13 ± 0.11	1.77 ± 0.15	1.75 ± 0.12	1.63 ± 0.10	1.80 ± 0.04	1.92 ± 0.12	1.90 ± 0.09	1.80 ± 0.10	1.75 ± 0.16	1.69 ± 0.06
150 µM Zn	1.89 ± 0.10	1.61 ± 0.10	1.76 ± 0.12	1.65 ± 0.11	1.75 ± 0.06	1.57 ± 0.17	1.78 ± 0.13	1.85 ± 0.06	1.62 ± 0.07	1.61 ± 0.09
Recovery	1.89 ± 0.10	1.69 ± 0.11	1.68 ± 0.11	1.96 ± 0.07*	2.30 ± 0.19×	1.95 ± 0.10	2.08 ± 0.14	2.21 ± 0.15*	2.14 ± 0.17	2.05 ± 0.11×
Manganese, µmol/g DW										
Roots										
Control	4.61 ± 1.00	6.08 ± 1.28	2.60 ± 0.33	3.22 ± 0.43	2.77 ± 0.63	4.99 ± 0.35	5.91 ± 1.44	3.52 ± 0.60	1.72 ± 0.39	4.37 ± 1.37
150 µM Zn	1.09 ± 0.06×	0.64 ± 0.06×	0.55 ± 0.08*	0.54 ± 0.05×	0.71 ± 0.07×	0.53 ± 0.04×	0.66 ± 0.09×	0.45 ± 0.03×	0.34 ± 0.04×	0.36 ± 0.03×
Recovery	1.09 ± 0.06×	2.23 ± 0.24×	1.92 ± 0.27	3.77 ± 0.33	4.35 ± 0.69	2.58 ± 0.40*	2.58 ± 0.40	1.61 ± 0.29*	2.72 ± 0.55	1.64 ± 0.27
Hypocotyls										
Control	0.69 ± 0.07	1.04 ± 0.09	0.86 ± 0.05	1.24 ± 0.11	0.74 ± 0.04	0.83 ± 0.07	0.76 ± 0.07	0.73 ± 0.04	0.73 ± 0.08	0.82 ± 0.06
150 µM Zn	0.73 ± 0.08	0.68 ± 0.06*	0.57 ± 0.02*	0.65 ± 0.04*	0.72 ± 0.06	0.52 ± 0.05*	0.61 ± 0.09	0.51 ± 0.05*	0.42 ± 0.05*	0.49 ± 0.07×
Recovery	0.73 ± 0.08	1.22 ± 0.06	0.77 ± 0.08	0.95 ± 0.07	0.48 ± 0.03*	0.67 ± 0.04	0.68 ± 0.07	0.73 ± 0.05	0.60 ± 0.07	0.71 ± 0.10

Cotyledons										
Control	12.3 ± 0.67	14.2 ± 0.99	12.9 ± 0.55	13.4 ± 1.01	12.1 ± 0.79	10.8 ± 1.29	12.1 ± 1.24	12.8 ± 1.60	11.7 ± 0.90	14.3 ± 1.93
150 μM Zn	9.79 ± 0.51*	9.45 ± 0.33*	9.43 ± 0.21*	8.92 ± 0.36*	9.61 ± 0.68*	9.17 ± 1.92	8.80 ± 1.30	9.09 ± 0.61	9.49 ± 1.23	8.81 ± 0.81*
Recovery	9.79 ± 0.51*	11.5 ± 0.55	9.62 ± 0.99*	9.92 ± 1.22	9.46 ± 1.51	8.62 ± 0.87	10.46 ± 1.46	9.07 ± 1.09	9.28 ± 1.01	10.4 ± 0.78
Needles										
Control	5.77 ± 0.27	6.21 ± 0.18	6.31 ± 0.26	5.61 ± 0.36	6.01 ± 0.37	6.25 ± 0.23	6.47 ± 0.39	6.13 ± 0.17	5.87 ± 0.32	6.34 ± 0.50
150 μM Zn	3.01 ± 0.11*	3.21 ± 0.08*	2.94 ± 0.06×	2.74 ± 0.12*	2.63 ± 0.07×	2.73 ± 0.16*	2.77 ± 0.12×	2.76 ± 0.07×	2.51 ± 0.15*	2.27 ± 0.11×
Recovery	3.01 ± 0.11*	3.10 ± 0.08*	3.15 ± 0.09*	3.38 ± 0.12×	3.50 ± 0.19*	3.67 ± 0.12*	3.80 ± 0.21*	3.83 ± 0.12*	4.57 ± 0.33*	4.34 ± 0.41*
Copper, nmol/g DW										
Roots										
Control	306.9 ± 22.2	224.3 ± 32.9	296.6 ± 17.8	278.1 ± 13.9	353.9 ± 31.5	278.7 ± 18.9	315.4 ± 29.8	331.9 ± 15.6	272.9 ± 7.25	323.0 ± 14.8
150 μM Zn	300.7 ± 11.7	235.7 ± 17.2	275.2 ± 14.9	274.3 ± 15.0	290.2 ± 21.0	270.2 ± 23.8	270.8 ± 12.8	397.4 ± 18.4	310.0 ± 19.5	354.4 ± 12.6
Recovery	300.7 ± 11.7	303.0 ± 13.9	277.3 ± 12.1	338.7 ± 9.9*	352.0 ± 15.1	299.7 ± 18.9	283.1 ± 13.1	350.3 ± 9.37	358.8 ± 22.1*	429.3 ± 14.1*
Hypocotyls										
Control	145.6 ± 15.1	163.1 ± 22.5	104.0 ± 11.2	167.8 ± 17.5	137.7 ± 32.0	100.6 ± 5.65	125.1 ± 15.3	146.6 ± 16.5	ND	125.6 ± 8.32
150 μM Zn	118.1 ± 7.63	111.9 ± 6.99	124.2 ± 3.12	79.4 ± 5.76×	94.8 ± 5.97	80.3 ± 6.78	82.8 ± 3.85×	124.5 ± 10.5	ND	129.1 ± 6.64
Recovery	118.1 ± 7.63	135.2 ± 8.70	147.7 ± 3.20*	83.4 ± 4.36×	120.7 ± 9.12	132.9 ± 4.82*	63.1 ± 12.0*	119.2 ± 16.8	ND	128.7 ± 10.8
Cotyledons										
Control	123.2 ± 6.27	93.2 ± 5.81	66.6 ± 9.99	108.6 ± 12.4	124.1 ± 7.00	91.1 ± 8.52	103.9 ± 17.1	93.2 ± 3.60	91.2 ± 7.10	139.3 ± 9.82
150 μM Zn	98.3 ± 15.3	74.5 ± 4.55*	40.1 ± 7.64	161.5 ± 17.1	151.8 ± 37.8	150.4 ± 13.2*	163.6 ± 36.9	97.7 ± 12.0	102.5 ± 20.6	122.8 ± 14.0
Recovery	98.3 ± 15.3	73.5 ± 4.10*	89.4 ± 9.15	124.6 ± 7.44	107.2 ± 7.71	124.7 ± 5.64*	156.1 ± 15.9	113.8 ± 27.1	100.6 ± 11.6	100.6 ± 6.5*
Needles										
Control	139.3 ± 3.83	142.4 ± 3.69	125.6 ± 4.33	123.8 ± 3.60	134.2 ± 5.10	114.3 ± 5.92	132.3 ± 8.99	125.6 ± 2.35	112.4 ± 5.26	131.7 ± 4.85
150 μM Zn	141.6 ± 2.51	132.0 ± 2.49*	120.5 ± 4.32	109.2 ± 5.23*	124.2 ± 5.27	109.7 ± 3.86	110.6 ± 4.05*	127.3 ± 7.52	112.7 ± 4.77	114.5 ± 3.69*
Recovery	141.6 ± 2.51	134.5 ± 1.41	116.9 ± 2.34	117.8 ± 3.26	141.5 ± 6.73	114.5 ± 3.19	115.4 ± 2.11	121.8 ± 4.71	112.3 ± 3.06	131.7 ± 5.59

Pairwise comparisons of the means with controls at corresponding time points were performed using Student's *t*-test for normally distributed data (significant differences at $p < 0.05$ denoted by asterisk (*)) or Mann-Whitney rank sum test when the *t*-test was not applicable (significant differences at $p < 0.05$ denoted by multiplication symbols (×)). ND – no data.
