# Supplementary material for: Recovery of Scots Pine Seedlings from Long-Term Zinc Toxicity
Source: Plants (Basel). 2024 Aug 11;13(16):2227. doi: 10.3390/plants13162227 (PMC11359686; doi:10.3390/plants13162227)
Supplement: Supplementary file 1 [file plants-13-02227-s001.zip › Table S3.pdf]

Table S3. The dynamics of nutrient amount in the organs of Scots pine seedlings throughout the experiment.

Variant	Initial point	Day of the experiment								
		1st	3rd	5th	7th	10th	14th	17th	21st	28th
Zinc, nmol										
Roots										
Control	18.7 ± 1.99	18.0 ± 2.74	14.3 ± 1.50	21.6 ± 2.13	34.2 ± 5.75	29.4 ± 4.14	30.8 ± 4.09	31.7 ± 2.71	32.0 ± 3.41	76.2 ± 19.2
150 μM Zn	299.6 ± 21.3×	242.6 ± 29.7×	306.8 ± 21.7×	266.1 ± 33.5×	221.7 ± 22.4*	337.2 ± 31.8×	397.4 ± 61.4×	430.0 ± 33.4×	443.9 ± 36.0×	843.4±239.5×
Recovery	299.6 ± 21.3×	268.2 ± 31.0×	202.4 ± 27.6×	213.9 ± 16.2×	167.1 ± 18.2×	168.4 ± 18.4×	161.2 ± 16.4×	158.0 ± 17.4×	151.9 ± 16.3×	176.3 ± 18.6×
Hypocotyls										
Control	5.86 ± 0.50	6.93 ± 1.18	8.10 ± 3.40	5.42 ± 0.97	9.12 ± 0.63	5.44 ± 0.82	6.37 ± 0.25	9.17 ± 1.17	6.63 ± 0.36	12.0 ± 1.63
150 μM Zn	44.0 ± 4.65*	33.1 ± 1.74*	41.7 ± 2.07×	37.2 ± 5.09×	66.1 ± 15.4×	42.6 ± 4.20×	66.7 ± 12.1×	79.6 ± 12.3×	66.6 ± 6.49×	90.8 ± 14.0×
Recovery	44.0 ± 4.65*	48.5 ± 5.78*	39.6 ± 11.1×	49.9 ± 9.71×	35.2 ± 3.30×	32.4 ± 2.53*	34.2 ± 1.52*	47.9 ± 9.25×	32.8 ± 3.51×	36.5 ± 4.77*
Cotyledons										
Control	5.74 ± 0.29	6.85 ± 0.36	8.10 ± 1.64	10.1 ± 1.63	7.80 ± 1.11	9.25 ± 1.07	7.18 ± 1.14	5.23 ± 0.47	7.60 ± 0.48	8.01 ± 1.50
150 μM Zn	38.9 ± 2.43*	36.9 ± 1.31*	38.8 ± 3.44*	37.3 ± 3.30*	40.6 ± 5.83×	46.2 ± 1.32*	41.8 ± 4.48*	37.4 ± 3.77×	42.8 ± 4.51×	51.7 ± 4.91*
Recovery	38.9 ± 2.43*	41.8 ± 3.88×	34.9 ± 3.36*	49.1 ± 4.43*	31.4 ± 2.73*	45.2 ± 6.52×	44.4 ± 2.19×	36.9 ± 2.84×	43.7 ± 3.28×	39.2 ± 2.13*
Needles										
Control	34.1 ± 2.02	34.1 ± 3.80	37.4 ± 3.59	45.5 ± 7.08	61.7 ± 6.11	59.6 ± 3.63	69.2 ± 7.86	66.8 ± 2.63	71.1 ± 6.18	140.9 ± 22.9
150 μM Zn	210.1 ± 17.1×	205.2 ± 12.3×	260.1 ± 33.4×	220.3 ± 9.99*	264.5 ± 21.5*	335.6 ± 28.1×	358.3 ± 45.5×	534.6 ± 48.3×	502.6 ± 62.3×	686.8±160.5×
Recovery	210.1 ± 17.1×	250.7 ± 23.0×	232.3 ± 33.9×	274.0 ± 16.0*	224.6 ± 16.9*	340.8 ± 56.1×	294.3 ± 29.7×	319.8 ± 23.0×	332.8 ± 36.2×	314.6 ± 27.4*
Magnesium, μmol										
Roots										
Control	0.35 ± 0.01	0.30 ± 0.03	0.25 ± 0.02	0.35 ± 0.03	0.55 ± 0.06	0.44 ± 0.02	0.50 ± 0.03	0.63 ± 0.07	0.57 ± 0.05	1.05 ± 0.12
150 μM Zn	0.16 ± 0.02*	0.12 ± 0.01×	0.12 ± 0.01*	0.13 ± 0.01*	0.15 ± 0.02*	0.19 ± 0.03*	0.23 ± 0.03*	0.33 ± 0.03*	0.29 ± 0.03*	0.48 ± 0.12*
Recovery	0.16 ± 0.02*	0.13 ± 0.01×	0.15 ± 0.02×	0.18 ± 0.01×	0.22 ± 0.02×	0.29 ± 0.05*	0.36 ± 0.05*	0.59 ± 0.04	0.46 ± 0.05	0.87 ± 0.10
Hypocotyls										
Control	0.24 ± 0.01	0.24 ± 0.01	0.28 ± 0.01	0.32 ± 0.04	0.48 ± 0.03	0.35 ± 0.03	0.35 ± 0.02	0.43 ± 0.05	0.38 ± 0.04	0.56 ± 0.07
150 μM Zn	0.19 ± 0.02	0.15 ± 0.01*	0.20 ± 0.01*	0.19 ± 0.02*	0.29 ± 0.07*	0.28 ± 0.05	0.30 ± 0.04	0.22 ± 0.03*	0.23 ± 0.03*	0.30 ± 0.03*
Recovery	0.19 ± 0.02	0.20 ± 0.02	0.19 ± 0.02*	0.27 ± 0.04	0.23 ± 0.02*	0.30 ± 0.03	0.37 ± 0.03	0.36 ± 0.02	0.37 ± 0.04	0.40 ± 0.03
Cotyledons										
Control	0.67 ± 0.02	0.69 ± 0.04	0.69 ± 0.02	0.73 ± 0.05	0.95 ± 0.07	0.76 ± 0.05	0.83 ± 0.10	0.68 ± 0.03	0.87 ± 0.05	0.80 ± 0.06
150 μM Zn	0.67 ± 0.03	0.64 ± 0.04	0.67 ± 0.03	0.57 ± 0.03*	0.66 ± 0.05*	0.64 ± 0.03	0.71 ± 0.03	0.67 ± 0.05	0.61 ± 0.03*	0.69 ± 0.04
Recovery	0.67 ± 0.03	0.65 ± 0.03	0.52 ± 0.03*	0.73 ± 0.05	0.55 ± 0.04*	0.73 ± 0.08	0.65 ± 0.04	0.69 ± 0.02	0.66 ± 0.03*	0.71 ± 0.04
Needles										
Control	2.52 ± 0.10	2.50 ± 0.25	2.69 ± 0.28	3.35 ± 0.51	4.93 ± 0.50	4.50 ± 0.32	5.26 ± 0.41	5.83 ± 0.38	5.31 ± 0.54	10.01 ± 1.36
150 μM Zn	1.33 ± 0.10*	1.28 ± 0.09×	1.63 ± 0.11*	1.58 ± 0.12×	1.82 ± 0.14×	2.19 ± 0.36*	2.64 ± 0.39×	3.02 ± 0.28*	2.88 ± 0.39*	4.34 ± 1.14×
Recovery	1.33 ± 0.10*	1.51 ± 0.13×	1.49 ± 0.15*	1.76 ± 0.12×	1.82 ± 0.14×	2.85 ± 0.37*	3.24 ± 0.37*	4.21 ± 0.44*	4.50 ± 0.56	6.06 ± 0.68*
Iron, nmol										
Roots										
Control	345.3 ± 47.5	352.5 ± 33.0	304.1 ± 31.6	410.4 ± 18.7	471.9 ± 26.7	639.6 ± 51.3	1089.7 ± 76.0	1300.1±191.4	1292.8±139.5	4399.8±688.7
150 μM Zn	187.8 ± 26.8*	236.0 ± 34.0	254.1 ± 23.1	288.2 ± 20.2*	272.2 ± 19.7*	458.9 ± 46.0*	655.8 ± 133.1*	805.7±95.0*	1150.9±128.7	2548.7±695.9×
Recovery	187.8 ± 26.8*	208.1 ± 32.3×	188.5 ± 36.7*	324.5 ± 29.1*	231.9 ± 63.8×	579.3 ± 162.7	745.6 ± 146.4	1464.8±187.0	1312.9±121.8	3397.9±532.7
Hypocotyls										
Control	6.46 ± 1.25	4.87 ± 1.45	4.63 ± 1.10	7.93 ± 1.58	14.1 ± 3.03	14.2 ± 3.20	2.60 ± 0.32	8.98 ± 1.94	7.78 ± 1.71	20.5 ± 7.38
150 μM Zn	4.64 ± 0.63	5.94 ± 1.86	9.20 ± 0.52*	6.55 ± 1.89	8.58 ± 1.29	8.74 ± 1.18	5.84 ± 1.64	14.0 ± 2.68	4.83 ± 2.76	17.3 ± 4.77
Recovery	4.64 ± 0.63	7.15 ± 0.94	5.10 ± 0.58	10.3 ± 4.09	7.22 ± 3.63	5.62 ± 2.12	4.38 ± 0.75	15.6 ± 3.81	4.97 ± 1.24	11.2 ± 1.10
Cotyledons										
Control	6.46 ± 0.52	9.67 ± 1.65	7.64 ± 2.36	8.63 ± 1.21	21.3 ± 6.34	10.1 ± 2.73	12.0 ± 3.31	9.20 ± 3.14	10.7 ± 1.24	14.2 ± 6.10
150 μM Zn	7.19 ± 1.60	6.40 ± 0.54	3.60 ± 1.12	7.22 ± 1.76	5.92 ± 1.92*	3.67 ± 0.85	8.78 ± 2.10	5.47 ± 0.70	17.5 ± 4.10	6.50 ± 1.81
Recovery	7.19 ± 1.60	8.03 ± 0.47	5.13 ± 1.32	6.60 ± 1.45	9.08 ± 3.23	5.70 ± 1.66	7.74 ± 0.93	13.2 ± 1.90	13.5 ± 3.22	9.78 ± 1.91
Needles										
Control	69.5 ± 4.89	55.8 ± 8.44	65.9 ± 10.7	72.9 ± 13.9	105.1 ± 5.74	112.6 ± 5.30	123.3 ± 10.9	130.9 ± 11.8	119.1 ± 10.6	211.0 ± 39.6
150 μM Zn	43.6 ± 3.74*	39.1 ± 6.14	53.9 ± 4.87	48.1 ± 6.58	58.3 ± 7.95*	64.3 ± 8.00*	81.9 ± 11.1*	97.5 ± 9.07*	94.3 ± 11.5	122.2 ± 25.3×
Recovery	43.6 ± 3.74*	50.4 ± 8.08	47.8 ± 7.14	65.0 ± 5.82	65.6 ± 8.64*	87.7 ± 12.9	104.5 ± 9.6	123.5 ± 8.1	128.5 ± 15.5	155.2 ± 12.4
Manganese, nmol										
Roots										
Control	29.5 ± 5.57	31.9 ± 5.77	15.6 ± 3.39	24.1 ± 2.56	29.5 ± 7.72	44.2 ± 3.67	69.7 ± 18.0	44.1 ± 6.10	21.3 ± 4.99	106.6 ± 45.8
150 μM Zn	5.33 ± 0.46×	2.80 ± 0.34×	3.03 ± 0.41×	2.66 ± 0.23×	3.50 ± 0.50×	3.94 ± 0.31×	5.94 ± 1.41×	4.80 ± 0.46×	4.13 ± 0.42×	5.61 ± 1.44×
Recovery	5.33 ± 0.46×	9.70 ± 0.90×	9.73 ± 1.16	22.9 ± 1.62	21.6 ± 2.96	20.7 ± 4.69*	23.7 ± 2.62	19.2 ± 3.54*	29.3 ± 6.91	27.1 ± 5.06
Hypocotyls										
Control	2.74 ± 0.22	4.05 ± 0.61	3.90 ± 0.41	5.86 ± 0.29	4.83 ± 0.25	5.20 ± 0.37	5.22 ± 0.53	5.51 ± 0.37	5.39 ± 0.53	10.3 ± 1.81
150 μM Zn	2.68 ± 0.39	2.06 ± 0.21×	2.20 ± 0.08×	2.41 ± 0.23*	3.19 ± 0.60*	2.66 ± 0.26*	3.55 ± 0.57	3.53 ± 0.40*	3.01 ± 0.34*	4.11 ± 0.65*
Recovery	2.68 ± 0.39	3.96 ± 0.23	2.75 ± 0.33	4.22 ± 0.30*	1.75 ± 0.12*	3.82 ± 0.47*	4.44 ± 0.28	4.60 ± 0.30	4.11 ± 0.59	6.51 ± 0.95

Cotyledons										
Control	48.4 ± 3.09	54.4 ± 2.90	49.4 ± 1.18	56.1 ± 5.53	61.7 ± 5.02	49.6 ± 8.05	51.3 ± 5.93	54.8 ± 6.69	54.8 ± 5.88	71.7 ± 12.6
150 μM Zn	39.4 ± 1.97*	32.5 ± 3.20*	34.4 ± 1.71*	27.8 ± 2.11*	35.2 ± 3.81*	37.6 ± 9.62	30.5 ± 4.22*	36.5 ± 2.59*	36.5 ± 6.56	37.9 ± 4.03×
Recovery	39.4 ± 1.97*	41.3 ± 1.62*	32.5 ± 2.50*	35.0 ± 3.33*	33.4 ± 8.72	33.3 ± 3.32	42.8 ± 5.90	34.8 ± 3.32*	34.2 ± 4.08*	44.4 ± 6.04
Needles										
Control	187.8 ± 9.52	195.1 ± 22.5	237.7 ± 37.7	240.5 ± 30.9	355.6 ± 36.2	370.8 ± 24.9	420.6 ± 39.4	443.2 ± 27.8	403.2 ± 33.0	786.4 ± 163.7
150 μM Zn	69.2 ± 5.11*	76.6 ± 8.54*	90.0 ± 5.50×	78.8 ± 7.41×	85.6 ± 8.90×	111.6 ± 9.46×	126.1 ± 13.1×	143.8 ± 10.1×	144.6 ± 16.0×	180.0 ± 43.3×
Recovery	69.2 ± 5.11*	89.4 ± 10.5*	90.0 ± 13.7*	111.2 ± 7.4×	100.8 ± 13.3×	166.5 ± 23.1*	192.9 ± 19.1×	217.2 ± 16.1*	269.9 ± 27.1*	328.2 ± 36.4×
Copper, nmol										
Roots										
Control	2.01 ± 0.08	1.26 ± 0.26	1.64 ± 0.10	2.19 ± 0.25	3.65 ± 0.48	2.46 ± 0.16	3.52 ± 0.41	4.39 ± 0.52	3.54 ± 0.26	6.76 ± 1.10
150 μM Zn	1.47 ± 0.11*	1.04 ± 0.13	1.53 ± 0.11	1.37 ± 0.08×	1.43 ± 0.16×	2.02 ± 0.16	2.32 ± 0.32*	4.20 ± 0.30	3.85 ± 0.41	5.71 ± 1.49
Recovery	1.47 ± 0.11*	1.39 ± 0.19	1.50 ± 0.27	2.12 ± 0.10	1.86 ± 0.26×	2.42 ± 0.38	2.76 ± 0.29	4.25 ± 0.25	3.68 ± 0.34	7.19 ± 0.76
Hypocotyls										
Control	0.18 ± 0.06	0.65 ± 0.13	0.48 ± 0.07	0.81 ± 0.09	0.87 ± 0.15	0.64 ± 0.04	0.86 ± 0.12	1.09 ± 0.08	ND	1.56 ± 0.25
150 μM Zn	0.42 ± 0.03*	0.34 ± 0.03	0.48 ± 0.02	0.29 ± 0.02*	0.41 ± 0.05*	0.41 ± 0.02*	0.48 ± 0.04*	0.85 ± 0.07*	ND	1.18 ± 0.29
Recovery	0.42 ± 0.03*	0.44 ± 0.03	0.53 ± 0.04	0.37 ± 0.03*	0.45 ± 0.05*	0.77 ± 0.11	0.40 ± 0.11*	0.74 ± 0.10*	ND	1.18 ± 0.09
Cotyledons										
Control	0.48 ± 0.03	0.37 ± 0.04	0.26 ± 0.04	0.47 ± 0.08	0.63 ± 0.05	0.40 ± 0.04	0.45 ± 0.08	0.40 ± 0.02	0.42 ± 0.04	0.68 ± 0.06
150 μM Zn	0.40 ± 0.06	0.26 ± 0.03	0.14 ± 0.02	0.49 ± 0.03	0.54 ± 0.11	0.61 ± 0.08*	0.58 ± 0.14	0.39 ± 0.05	0.37 ± 0.08	0.55 ± 0.09
Recovery	0.40 ± 0.06	0.27 ± 0.01	0.31 ± 0.04	0.45 ± 0.03	0.36 ± 0.04*	0.50 ± 0.06	0.65 ± 0.09	0.43 ± 0.08	0.37 ± 0.04	0.41 ± 0.02×
Needles										
Control	4.57 ± 0.30	4.45 ± 0.46	4.63 ± 0.58	5.34 ± 0.71	7.95 ± 0.73	6.72 ± 0.34	8.64 ± 0.89	9.06 ± 0.50	7.70 ± 0.56	16.2 ± 2.72
150 μM Zn	3.26 ± 0.23*	3.13 ± 0.34*	3.71 ± 0.30	3.22 ± 0.43*	4.00 ± 0.37×	4.53 ± 0.45*	5.23 ± 0.84*	6.69 ± 0.72*	6.52 ± 0.79	9.36 ± 2.47×
Recovery	3.26 ± 0.23*	3.90 ± 0.46	3.31 ± 0.45	3.92 ± 0.36	4.00 ± 0.39*	5.12 ± 0.62*	5.91 ± 0.62*	6.98 ± 0.64*	6.80 ± 0.82	10.3 ± 1.22*

Pairwise comparisons of the means with controls at corresponding time points were performed using Student's *t*-test for normally distributed data (significant differences at $p < 0.05$ denoted by asterisk (*)) or Mann-Whitney rank sum test when the *t*-test was not applicable (significant differences at $p < 0.05$ denoted by multiplication symbols (×)). ND – no data.
