# Supplementary material for: Recovery of Scots Pine Seedlings from Long-Term Zinc Toxicity
Source: Plants (Basel). 2024 Aug 11;13(16):2227. doi: 10.3390/plants13162227 (PMC11359686; doi:10.3390/plants13162227)
Supplement: Supplementary file 1 [file plants-13-02227-s001.zip › Table S4.pdf]

Table S4. Results of 2-way ANOVA describing the dependence of the dynamics of nutrient content in the organs of Scots pine seedlings on experimental variants and duration of the experiment.

Parameter	Roots			Hypocotyl			Cotyledons			Needles		
	V	T	V × T	V	T	V × T	V	T	V × T	V	T	V × T
Zn content			•			•	•	•	ns			•
Mg content			•	•	•	ns			•			•
Fe content	•	•	ns	ns	•	ns	ns	•	ns	•	•	ns
Mn content			•			•	•	ns	ns			•
Cu content			•			•			•	•	•	ns

The significance of the variant (V), sampling time (T), and variant × time (V × T) interaction were calculated using 2-way ANOVA ($p < 0.05$), with a circle (•) indicating significant differences and "ns" indicating no significant differences.
