# Supplementary material for: Recovery of Scots Pine Seedlings from Long-Term Zinc Toxicity
Source: Plants (Basel). 2024 Aug 11;13(16):2227. doi: 10.3390/plants13162227 (PMC11359686; doi:10.3390/plants13162227)
Supplement: Supplementary file 1 [file plants-13-02227-s001.zip › Table S5.pdf]

Table S5. Results of 2-way ANOVA describing the dependence of the photosynthetic pigment content in the basal and top needles of Scots pine seedlings on experimental variants and duration of the experiment.

**Parameter**	**Basal needles**			**Top needles**		
	Variant	Time	Variant × Time	Variant	Time	Variant × Time
Chlorophyll *a*	•	•	ns	•	ns	ns
Chlorophyll *b*			•	•	ns	ns
Carotenoids	•	•	ns	ns	ns	ns
Chl *a* / Chl *b*	ns	•	ns	ns	•	ns
Car / Chls	ns	•	ns	ns	•	ns

Circle (•) indicate significant differences at $p < 0.05$ and “ns” indicate no significant differences.
