# Supplementary material for: Recovery of Scots Pine Seedlings from Long-Term Zinc Toxicity
Source: Plants (Basel). 2024 Aug 11;13(16):2227. doi: 10.3390/plants13162227 (PMC11359686; doi:10.3390/plants13162227)
Supplement: Supplementary file 1 [file plants-13-02227-s001.zip › Table S6.pdf]

Table S6. The dynamics of low-molecular-weight antioxidants and lignin content in the organs of Scots pine seedlings during the experiment.

Variant	Initial point	Day of the experiment								
		1st	3rd	5th	7th	10th	14th	17th	21st	28th
TEAC, μmol of Trolox/g DW										
Roots										
Control	328.2 ± 26.1	348.9 ± 37.9	313.1 ± 25.5	364.8 ± 20.9	397.4 ± 34.8	358.2 ± 35.1	338.3 ± 13.0	360.0 ± 10.3	372.9 ± 32.8	357.4 ± 20.3
150 μM Zn	290.4 ± 14.2	227.2 ± 13.1	269.0 ± 16.2	198.6 ± 36.1*	226.2 ± 9.47*	212.5 ± 8.23*	276.6 ± 13.6*	295.1 ± 36.0	293.0 ± 19.8	273.0 ± 12.5*
Recovery	290.4 ± 14.2	233.9 ± 39.0	223.9 ± 20.7*	227.4 ± 9.7*	198.9 ± 24.9*	273.9 ± 24.2	334.6 ± 20.4	304.7 ± 14.3*	345.1 ± 18.6	288.4 ± 15.7×
Hypocotyls										
Control	106.7 ± 8.14	94.3 ± 2.41	86.8 ± 4.75	81.7 ± 5.31	88.3 ± 2.65	60.0 ± 1.35	54.8 ± 1.29	60.9 ± 3.11	53.3 ± 3.90	54.6 ± 2.67
150 μM Zn	112.8 ± 10.7	99.8 ± 18.9	100.3 ± 16.6	76.8 ± 5.16	74.0 ± 0.69*	61.3 ± 6.57	61.5 ± 4.60	67.2 ± 2.13	77.0 ± 8.03×	78.8 ± 6.60×
Recovery	112.8 ± 10.7	92.8 ± 0.67	87.6 ± 7.93	85.0 ± 9.30	67.5 ± 7.84*	57.6 ± 4.16	73.3 ± 1.49*	70.3 ± 3.44	61.8 ± 2.59	63.5 ± 3.66
Cotyledons										
Control	174.7 ± 7.63	239.7 ± 12.2	182.8 ± 6.18	186.4 ± 13.3	193.7 ± 7.10	215.9 ± 6.11	211.6 ± 7.29	233.9 ± 7.33	245.8 ± 9.73	209.9 ± 13.2
150 μM Zn	230.6 ± 17.2*	217.4 ± 13.4	213.4 ± 5.4*	204.7 ± 9.21	206.3 ± 5.28	222.0 ± 4.77	273.4 ± 5.63*	234.6 ± 9.12	262.9 ± 5.94	262.1 ± 18.5*
Recovery	230.6 ± 17.2*	283.8 ± 17.5	234.5 ± 19.3*	223.8 ± 8.62	218.9 ± 3.33*	265.2 ± 8.08*	223.0 ± 8.38	224.6 ± 14.0	248.5 ± 19.1	205.2 ± 6.97
Needles										
Control	218.3 ± 15.0	237.0 ± 13.2	196.3 ± 6.93	217.3 ± 11.2	206.0 ± 5.02	185.6 ± 6.78	191.9 ± 9.75	207.6 ± 8.12	175.5 ± 5.10	174.3 ± 5.78
150 μM Zn	248.5 ± 12.0	229.2 ± 11.7	264.4 ± 19.0*	246.1 ± 9.68	208.9 ± 5.69	245.1 ± 9.03*	257.0 ± 7.56*	233.2 ± 6.57*	216.5 ± 7.42*	275.6 ± 16.9*
Recovery	248.5 ± 12.0	259.6 ± 11.8	234.7 ± 4.38*	231.1 ± 10.9	204.5 ± 7.20	240.8 ± 5.21*	226.3 ± 13.5	247.4 ± 7.25*	217.0 ± 6.37*	206.1 ± 7.85*
GAE, mg/g DW										
Roots										
Control	29.4 ± 2.29	33.7 ± 5.04	28.3 ± 2.21	33.9 ± 2.55	38.2 ± 4.24	32.4 ± 3.57	29.5 ± 1.88	32.3 ± 1.37	28.5 ± 3.16	22.0 ± 2.83
150 μM Zn	25.8 ± 1.79	18.3 ± 1.75	24.8 ± 1.70	18.3 ± 3.97*	17.9 ± 0.83*	18.6 ± 0.98*	24.3 ± 2.02	21.2 ± 4.48*	23.5 ± 2.41	22.0 ± 1.32
Recovery	25.8 ± 1.79	22.0 ± 4.09	17.4 ± 2.10*	20.6 ± 0.93*	18.3 ± 3.94*	18.7 ± 1.42*	28.6 ± 2.40	28.2 ± 1.73	27.7 ± 0.96	20.3 ± 1.08
Hypocotyls										
Control	6.68 ± 1.30	7.53 ± 0.27	6.54 ± 0.17	4.94 ± 0.77	2.12 ± 0.70	2.53 ± 0.44	3.18 ± 0.19	3.79 ± 0.24	3.14 ± 0.34	3.35 ± 0.53
150 μM Zn	7.57 ± 1.24	5.84 ± 2.22	7.20 ± 1.33	4.68 ± 0.35	3.31 ± 0.37	3.63 ± 0.63	2.93 ± 0.46	3.67 ± 0.52	4.53 ± 0.75	5.99 ± 0.84*
Recovery	7.57 ± 1.24	6.39 ± 0.55	5.28 ± 0.71	5.30 ± 0.89	2.40 ± 0.57	2.90 ± 0.74	4.66 ± .15*	4.56 ± 0.49	4.30 ± 0.27	4.45 ± 0.43
Cotyledons										
Control	17.5 ± 1.12	18.4 ± 0.27	18.2 ± 0.54	19.2 ± 1.72	18.7 ± 0.50	18.0 ± 0.50	21.7 ± 0.60	22.1 ± 0.77	21.3 ± 1.06	20.7 ± 0.95
150 μM Zn	20.7 ± 0.84*	20.8 ± 0.63*	21.4 ± 0.59	20.8 ± 1.25	19.8 ± 0.47	22.8 ± 0.76*	21.8 ± 1.21	22.8 ± 0.68	26.1 ± 0.31*	24.5 ± 1.86
Recovery	20.7 ± 0.84*	22.6 ± 1.00*	21.0 ± 0.94*	22.1 ± 0.89	20.7 ± 0.73	25.9 ± 1.45*	22.9 ± 1.08	23.1 ± 1.89	22.3 ± 1.10	21.5 ± 0.66
Needles										
Control	12.9 ± 1.09	13.0 ± 0.67	11.5 ± 0.67	13.0 ± 0.81	11.9 ± 0.24	10.9 ± 0.46	10.8 ± 0.56	12.1 ± 0.60	10.6 ± 0.25	10.9 ± 0.31
150 μM Zn	14.8 ± 0.68	14.1 ± 0.69	14.9 ± 1.01	14.1 ± 0.36	12.6 ± 0.10×	14.3 ± 0.49*	14.7 ± 0.38*	13.8 ± 0.53	13.0 ± 0.50*	16.0 ± 0.61*
Recovery	14.8 ± 0.68	15.2 ± 0.51*	13.9 ± 0.17×	13.4 ± 0.43	12.1 ± 0.47	14.0 ± 0.09×	12.9 ± 0.68*	14.1 ± 0.39*	12.4 ± 0.37*	13.0 ± 0.52*
Catechins + proanthocyanidins, mg of (+)-catechin /g DW										
Roots										
Control	40.6 ± 3.69	46.7 ± 9.51	37.9 ± 4.03	46.9 ± 6.01	51.0 ± 7.34	47.7 ± 4.92	42.9 ± 2.74	48.6 ± 1.78	45.5 ± 3.93	35.2 ± 3.12
150 μM Zn	34.0 ± 2.80	27.0 ± 1.53	31.7 ± 2.50	25.2 ± 5.76	26.6 ± 1.85*	24.3 ± 1.14×	40.5 ± 6.53	35.1 ± 6.22	37.4 ± 3.03	32.6 ± 2.64
Recovery	34.0 ± 2.80	31.4 ± 6.08	24.8 ± 2.89*	28.3 ± 1.76*	24.8 ± 3.87*	29.1 ± 2.55*	49.5 ± 5.33	48.3 ± 2.96	42.8 ± 2.90	27.5 ± 2.43
Needles										
Control	4.57 ± 1.00	3.86 ± 0.19	2.83 ± 0.37	4.24 ± 0.34	3.38 ± 0.51	3.62 ± 0.43	3.99 ± .63	4.09 ± 0.92	3.06 ± 0.34	3.50 ± 0.42
150 μM Zn	7.62 ± 0.92*	5.14 ± 0.93	7.16 ± 1.52*	4.97 ± 0.51	4.65 ± 0.38	5.53 ± 0.23*	4.98 ± 0.28	5.81 ± 0.47	4.90 ± 0.90	6.18 ± 0.55*
Recovery	7.62 ± 0.92*	4.20 ± 0.29	5.03 ± 0.85*	3.29 ± 0.22*	3.86 ± 0.45	4.50 ± 0.35	3.84 ± 0.26	5.07 ± 1.29	3.21 ± 0.47	3.02 ± 0.28
Proanthocyanidins, mg of cyanidin /g DW										
Roots										
Control	11.5 ± 0.32	ND	ND	ND	ND	12.2 ± 0.52	ND	ND	13.7 ± 0.98	12.0 ± 0.74
150 μM Zn	9.37 ± 0.57*	ND	ND	ND	ND	8.34 ± 0.59*	ND	ND	10.2 ± 0.45*	7.94 ± 0.26*
Recovery	9.37 ± 0.57*	ND	ND	ND	ND	11.2 ± 0.68	ND	ND	12.0 ± 0.97	9.40 ± 0.94
Needles										
Control	1.68 ± 0.26	1.76 ± 0.13	1.12 ± 0.09	2.55 ± 0.58	1.64 ± 0.11	1.41 ± 0.07	1.61 ± 0.06	1.58 ± 0.26	1.10 ± 0.07	1.46 ± 0.25
150 μM Zn	2.39 ± 0.30	1.88 ± 0.37	2.40 ± 0.43×	2.03 ± 0.18	1.52 ± 0.06	2.52 ± 0.42×	3.07 ± 0.29×	2.44 ± 0.29	2.27 ± 0.31*	4.01 ± 0.71*
Recovery	2.39 ± 0.30	2.66 ± 0.39	2.24 ± 0.28*	1.93 ± 0.26	1.75 ± 0.12	2.86 ± 0.44×	1.91 ± 0.23*	2.60 ± 0.37	1.39 ± 0.10*	2.24 ± 0.20
Lignin, mg/g DW										
Roots										
Control	105.0 ± 5.19	ND	ND	ND	ND	118.1 ± 4.48	ND	ND	136.2 ± 5.64	133.1 ± 3.96
150 μM Zn	80.3 ± 3.52*	ND	ND	ND	ND	93.6 ± 5.03*	ND	ND	110.4 ± 4.66*	112.9 ± 4.86*
Recovery	80.3 ± 3.52*	ND	ND	ND	ND	101.1 ± 5.93*	ND	ND	107.0 ± 2.24*	115.2 ± 5.03*
Needles										
Control	30.7 ± 1.34	ND	31.3 ± 0.32	ND	ND	32.4 ± 0.68	34.5 ± 0.70	35.6 ± 0.85	38.5 ± 0.40	39.3 ± 0.68
150 μM Zn	31.9 ± 0.77	ND	31.7 ± 0.76	ND	ND	33.8 ± 0.76	33.2 ± 1.59	34.8 ± 1.17	35.0 ± 1.27×	36.5 ± 1.05*

Recovery	31.9 ± 0.77	ND	30.7 ± 0.83	ND	ND	31.8 ± 1.12	33.2 ± 0.93	34.2 ± 1.14	36.9 ± 0.71	35.7 ± 1.20×

Pairwise comparisons of the means with controls at corresponding time points were performed using Student's *t*-test for normally distributed data (significant differences at $p < 0.05$ denoted by asterisk (*)) or Mann-Whitney rank sum test when the *t*-test was not applicable (significant differences at $p < 0.05$ denoted by multiplication symbols (×)). ND – no data.
