# Supplementary material for: Recovery of Scots Pine Seedlings from Long-Term Zinc Toxicity
Source: Plants (Basel). 2024 Aug 11;13(16):2227. doi: 10.3390/plants13162227 (PMC11359686; doi:10.3390/plants13162227)
Supplement: Supplementary file 1 [file plants-13-02227-s001.zip › Table S7.pdf]

Table S7. Results of 2-way ANOVA describing the dependence of the dynamics of low-molecular-weight antioxidants and lignin content in the organs of Scots pine seedlings on experimental variants and duration of the experiment.

Parameter	Roots			Hypocotyl			Cotyledons			Needles		
	V	T	V × T	V	T	V × T	V	T	V × T	V	T	V × T
TEAC			•	ns	•	ns			•			•
GAE			•	ns	•	ns			•			•
Catechins + PAs			•	ND	ND	ND	ND	ND	ND	•	•	ns
PAs	•	•	ns	ND	ND	ND	ND	ND	ND			•
Lignin	•	•	ns	MD	ND	ND	ND	ND	ND	ns	•	ns

The significance of the variant (V), sampling time (T), and variant × time (V × T) interaction were calculated using 2-way ANOVA ($p < 0.05$), with a circle (•) indicating significant differences and "ns" indicating no significant differences.
